# Identification of *sucA,* Encoding β-Fructofuranosidase, in *Rhizopus microsporus*

**DOI:** 10.3390/microorganisms6010026

**Published:** 2018-03-13

**Authors:** Yoshitake Orikasa, Yuji Oda, Takuji Ohwada

**Affiliations:** Department of Life and Food Science, Obihiro University of Agriculture and Veterinary Medicine, Obihiro, Hokkaido 080-8555, Japan; yosori@obihiro.ac.jp (Y.Or.); yujioda@obihiro.ac.jp (Y.Od.)

**Keywords:** *Rhizopus microsporus*, β-fructofuranosidase, *sucA* gene, sucrose, fructooligosaccharides

## Abstract

*Rhizopus microsporus* NBRC 32995 was found to hydrolyze fructooligosaccharides (FOS), as well as sucrose, almost completely into monosaccharides through the production of sufficient amounts of organic acids, indicating that the complete hydrolysis of FOS was caused by the secretion of β-fructofuranosidase from fungal cells. Thus, the *sucA* gene, encoding a β-fructofuranosidase, was amplified by degenerate PCR, and its complete nucleotide sequence was determined. The total length of the *sucA* gene was 1590 bp, and the SucA protein of *R. microsporus* NBRC 32995 belonged to clade VIa, which also contains *Rhizopus delemar* and is closely related to Saccharomycotina, a subdivision of the Ascomycota.

## 1. Introduction

*Rhizopus* fungi are widely distributed in the environment, including in soil and on plant surfaces, and are known as “*kumonosukabi*” in Japan. Some *Rhizopus* strains have been recognized as harmful, causing infectious disease in humans and plants [1]. However, *Rhizopus* strains have a strong ability to convert sugar into organic acids, which are widely used as raw materials and additives in foods, cosmetics, and medicines. For example, *Rhizopus* strains are used to produce fermented foods in Asia [2], such as the traditional Indonesian soybean product, tempeh [3]. *Rhizopus oryzae* is one of the organic acid-producing *Rhizopus* species, and is classified into two groups: the lactic acid-producing *R. oryzae* (LA group) and the malic-fumaric acid-producing *Rhizopus delemar* (FMA group). These classifications are supported by genetic and phylogenetic analyses [4,5].

Several types of microbial enzymes are known to be involved in the degradation of sugars such as sucrose and fructooligosaccharides (FOS), and widely distributed in plants, yeasts, and bacteria [6]. For example, β-fructofuranosidase (EC3.2.1.26) hydrolyzes sugars from fructosides, but glucoamylase (EC3.2.1.3) hydrolyzes sugars from glucosides. In particular, β-fructofuranosidase is useful for the production of organic acids owing to its stability even at low pH levels [7,8]. Thus, the distribution and classification of enzymes with β-fructofuranosidase activity have been previously investigated among several strains of *R. oryzae* and *R. delemar*. The results showed that the *sucA* gene encoding β-fructofuranosidase was present in only a few strains of *R. delemar* [7,8], and its DNA sequence revealed that the *sucA* genes in *R. delemar* strains belonged to a group of fungi distinct from other zygomycetes, but closely related to ascomycetes [8]. It was reported that the SucA (i.e., β-fructofuranosidase) can be classified into seven clades based on its conserved amino acid sequence [9].

The *Rhizopus* filamentous fungi are classified into three groups, *Rhizopus stolonifer*, *R. oryzae* (including *R. delemar*), and *Rhizopus microsporus,* based on culture temperature, the sporangium, and the size of the sporangiophore [5,10,11]. Thus, in this study, *R. microsporus* and *R. stolonifer* strains were evaluated for the production of organic acids and ethanol as well as the distribution of the *sucA* gene. In particular, *R. microsporus* is known to exhibit an excellent ability to produce lactic acid and grow at high temperatures [12]. Therefore, the hydrolytic activity of the *R. microsporus* strain against both sucrose and fructooligosaccharides (FOS) for the production of organic acids was evaluated. Moreover, the *sucA* gene of *R. microsporus* was sequenced and its phylogenetic position was decided.

## 2. Materials and Methods

### 2.1. Strains and Culture Conditions

The following *Rhizopus* strains were obtained from the NITE Biological Research Center (Chiba, Japan): *Rhizopus microsporus* var. *chinensis* NBRC 4737, 4768, 31988; *R. microsporus* var. *microsporus* NBRC 32995, 32996; *R. microsporus* var. *rhizopodiformis* NBRC 32997; *R. microsporus* var. *tuberosus* NBRC 100014; *R. stolonifer* var. *lyococcus* NBRC 32998; *R. stolonifer* var. *stolonifer* NBRC 4781, 5411, 6188, 30816. Cells were grown on potato dextrose agar (Becton Dickinson Company, Franklin Lakes, NJ, USA), which was prepared horizontally in a standing test tube. Mycelia were collected and used to inoculate 100 mL of liquid medium composed of 10% sucrose or short-chain FOS (mixture of 1-kestose, nystose, and 1-fructofuranosyl-d-nystose) [13] as the carbon source, as well as 0.2% (NH_4_)_2_SO_4_, 0.065% KH_2_PO_4_, 0.025% MgSO_4_, and 2.5% CaCO_3_ in a 300 mL Erlenmeyer flask. Cultures were incubated at 30 °C with shaking (150 rpm).

### 2.2. Analyses of Organic Acids Production and Sugar Hydrolysis

A portion of each culture was withdrawn and, after centrifugation at 3000× *g* for 15 min at 4 °C, the amounts of organic acid in the supernatant was determined by a high-performance liquid chromatography system (LaChrom Elite, Hitachi High Technologies, Tokyo, Japan) equipped with a packed column (Shodex RSpak KC-811; Showa Denko Co., Tokyo, Japan) and ultraviolet (UV) monitor (SPD-10AVP, SIMADZU, Kyoto, Japan). The hydrolytic activities against sugars were analyzed by thin-layer chromatography (TLC) using a solvent system of 1-butanol:2-propanol:acetic acid:water (7:5:2:4, *v*/*v*). Each spot of sugar was visualized by spraying with anisaldehyde-sulfuric acid reagent according to the method of Watanabe and Oda [7]. 

### 2.3. Preparation of Cell-Free Extracts

Fungal cells were harvested by centrifugation at 3000× *g* for 5 min, and cell pellets were stored at −80 °C until use. Harvested cell pellets were washed with 0.1 M potassium phosphate buffer (pH 7.4) to remove residual medium components and resuspended in the same fresh buffer. The cell suspension was disrupted by sonication (pulse on, 3 s; pulse off, 5 s; power, 40%) on ice for 10 min using a model JY 92-IIN sonicator (Scientz, Ningbo, China) and centrifuged at 6000× *g* for 15 min at 4 °C. The supernatant was used as a cell-free extract for the following enzyme assays.

### 2.4. Enzyme Assays

Sucrose-hydrolyzing activity was determined according to the method of Oda and Tonomura [14]. Briefly, a portion of the cell-free extract was added to 100 mM acetate buffer (pH 5.0) containing 150 mM sucrose to a final volume of 0.25 mL. After incubation at 30 °C for 30 min, the reaction was stopped by the addition of 1.0% (*w*/*v*) 3,5-dinitrosalicylic acid reagent, and the amount of reducing sugar produced was determined by the method of Oda and Tonomura [14]. One unit was defined as the amount of enzyme that released 1 µmol of reducing sugar equivalent to glucose per minute under the above conditions.

### 2.5. Identification and Sequencing of sucA Gene

Chromosomal DNA was extracted from lyophilized cells of each strain according to the methods of Sone et al. [15] and purified using the ZR Fungal/Bacterial DNA kit (ZYMO Research, Irvine, CA, USA). Oligonucleotide primers (RD_dPCR_INV_F1, 5′-TGGATGAAYGAYCCIAAYGGIYTITTYTAYGA-3′; RD_dPCR_INV_R1, 5′-TAYTGCCARTTISWIGCCCAIGCIARNCC-3′) were designed by Primer3Plus using the conserved sequences of domains A and E in the SucA of *R. delemar* NBRC 4754 (AB701479), *Amylomyces rouxii* CBS 436.76 (AB701482), and *Schwanniomyces occidentalis* ATCC 26077 (ADN34605), respectively. Degenerate PCR was performed for the detection of the *sucA* gene under the following amplification conditions using a thermal cycler (T100™ Thermal Cycler; Bio-Rad, Hercules, CA, USA): denaturation at 96 °C for 1 min, annealing at 50 °C for 30 s, and extension at 72 °C for 2 min. The whole genome sequencing was performed with a next-generation sequencer (MiSeq system; Illumina, San Diego, CA, USA) using the paired-end mode (300 bp × 2) in combination with the software program Tablet for next-generation sequence assembly [16]. The DNA sequence of the *sucA* gene was identified using the BioEdit multiple sequence alignment editor (Ibis Biosciences, Carlsbad, CA, USA) and the BLAST database.

## 3. Results

### 3.1. Detection of β-Fructofuranosidase (sucA) Gene and Production of Organic Acids and Ethanol in Rhizopus Strains

Degenerate oligonucleotide primers were designed based on a conserved region closely related to the sequence of the fungal *sucA* gene, and PCR was performed using these degenerate primers. The presence/absence of *sucA* genes among the seven *R. microsporus* strains and five *R. stolonifera* strains tested is shown in Table 1. DNA amplification occurred only in *R. microsporus* NBRC 32995, indicating that among the *Rhizopus* strains tested, only this strain carries the *sucA* gene. The amplified DNA fragment was approximately 1.6 kbp according to gel electrophoresis.

The *Rhizopus* strains were incubated in liquid medium containing sucrose as a carbon source at 30 °C for 14 days, and the production levels of organic acids (malic acid, lactic acid, and fumaric acid) and ethanol were evaluated (Table 1). *R. microsporus* NBRC 32995 produced the highest levels of all organic acids and ethanol among all *R. microsporus* strains tested. In particular, the production of lactic acid was significantly higher than that of the other organic acids, reaching approximately 58.1 mg/mL of culture. Among *R. stolonifer* strains, the production of all organic acids and ethanol was the highest for *R. stolonifer* NBRC 4781, and the production level of lactic acid was significantly higher than those of the other organic acids. While the production level of lactic acid from *R. microsporus* NBRC 32995 was lower than those from *R. stolonifer* NBRC 4781 and *R. oryzae* NBRC 4785, only *R. microsporus* NBRC 32995 carries the *sucA* gene.

### 3.2. Analysis of Sucrose and FOS Hydrolysis by TLC

**Figure 1:**
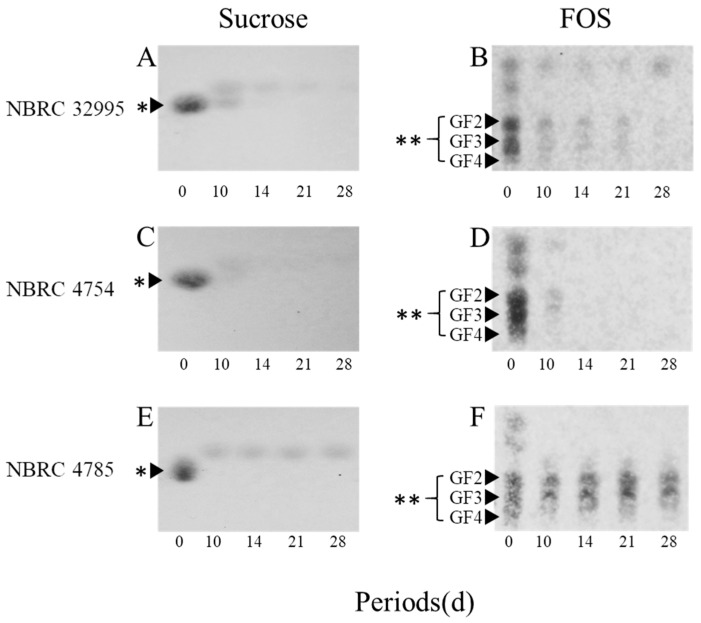
Thin-layer chromatography (TLC) analyses of residual sugars in media containing sucrose (**A**,**B**,**E**) or fructooligosaccharides (**B**,**D**,**F**) as the carbon source during the incubation of *R. microsporus* NBRC 32995 (**A**,**B**), *R. delemar* NBRC 4754 (**C**,**D**), and *R. oryzae* NBRC 4785 (**E**,**F**). A portion of each culture was withdrawn, and the residual sugars in the supernatant were separated by TLC. Spots corresponding to sucrose and fructooligosaccharides are indicated by single (*) and double asterisks (**), respectively. GF2, 1-kestose; GF3, nystose; GF4, 1-fructofuranosyl-d-nystose.

The hydrolysis of sucrose and FOS by *R. microsporus* NBRC 32995 was compared with those of *R. delemar* NBRC 4754 and *R. oryzae* NBRC 4785 (Figure 1). For all strains tested, according to TLC, the spot of sucrose almost disappeared, and new spots corresponding to monosaccharide emerged after 10 days of incubation, indicating that these strains have the ability to decompose sucrose into monosaccharides (Figure 1A,C,E). The spots corresponding to monosaccharides produced by the decomposition of sucrose mostly disappeared after 21 days of incubation for both *R. microsporus* NBRC 32995 and *R. delemar* NBRC 4754, but some monosaccharides remained up to 28 days of incubation for *R. oryzae* NBRC 4785 (Figure 1E). Moreover, for both *R. microsporus* NBRC 32995 and *R. delemar* NBRC 4754, almost all FOS spots were significantly reduced in intensity after 10 days and had completely disappeared after 28 days of incubation (Figure 1B,D). However, for *R. oryzae* NBRC 4785, FOS spots remained and their intensities were maintained to some extent up to 28 days of incubation (Figure 1F). These results demonstrate that *R. microsporus* NBRC 32995 as well as *R. delemar* NBRC 4754 have the ability to hydrolyze both sucrose and FOS into monosaccharides, indicating the complete hydrolysis of FOS by β-fructofuranosidase; however, *R. oryzae* NBRC 4785 did not show the ability to hydrolyze FOS.

### 3.3. Sucrose-Hydrolyzing Activity and pH in the Culture Medium

**Figure 2:**
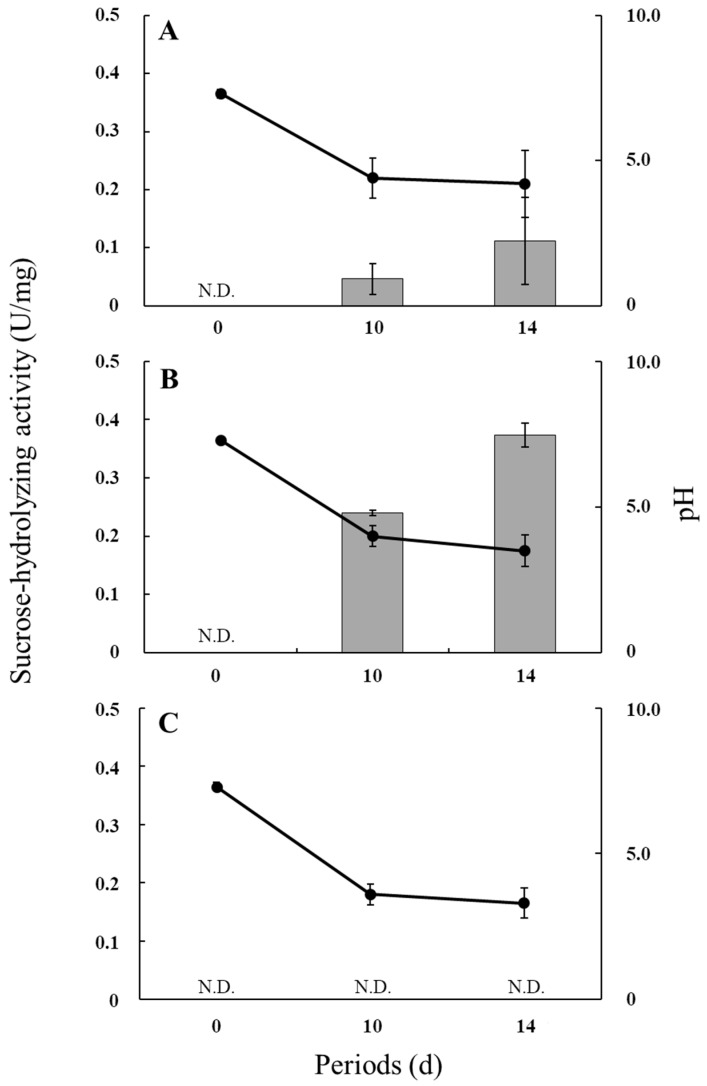
Sucrose-hydrolyzing activities and pH values of media during the incubation of *R. microsporus* NBRC 32995 (**A**); *R. delemar* NBRC 4754 (**B**); and *R. oryzae* NBRC 4785 (**C**). Cell-free extracts were prepared, and the sucrose-hydrolyzing activities (bars: U/mg protein) were measured. Values are the means of three replicates. N.D.: not detected.

Figure 2 shows the sucrose-hydrolyzing activities and culture pH values during the incubation of *R. microsporus* NBRC 32995, *R. delemar* NBRC 4754, and *R. oryzae* NBRC 4785. The pH values in all cultures decreased during incubation owing to the release of organic acids into the medium (Table 1). Under the condition of low pH, the sucrose-hydrolyzing activity of *R. microsporus* NBRC 32995 appeared to increase during incubation, with the level at 14 days being approximately twice that at 10 days (approximately 0.1 U/mg protein) (Figure 2A). *R. delemar* NBRC 4754 also exhibited sucrose-hydrolyzing activity that increased during incubation to approximately 0.37 U/mg protein at 14 days of incubation (Figure 2B), indicating that *R. microsporus* NBRC 32995 as well as *R. delemar* NBRC 4754 have the ability to hydrolyze sucrose even at a low pH. However, *R. oryzae* NBRC 4785 did not show sucrose-hydrolyzing activity at either 10 or 14 days of incubation under a low pH. The pH value in the *R. oryzae* NBRC 4785 culture was reduced to approximately 3.6 by day 10 of incubation (Figure 2C). Since the ability of glucoamylase to hydrolyze sucrose drops by approximately 90% at pH 3.5 [7], these results indicate that *R. oryzae* NBRC 4785 does not have β-fructofuranosidase activity capable of hydrolyzing sucrose under a low pH. These results support the finding that the *sucA* gene is present in both *R. microsporus* NBRC 32995 and *R. delemar* NBRC 4754 but not in *R. oryzae* NBRC 4785.

### 3.4. Sequencing and Phylogenetic Analysis of β-Fructofuranosidase (sucA) Gene

**Figure 3:**
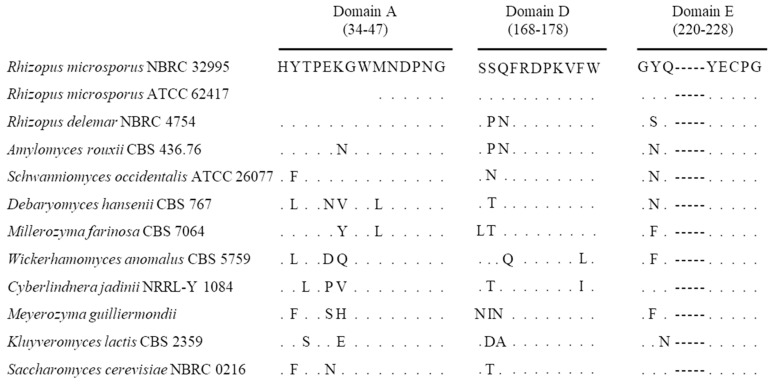
Conserved domains A, D, and E from microbial β-fructofuranosidases in clade VIa. Numbers in parentheses indicate positions in the enzyme from *R. oryzae*. Dots indicate identical amino acid residues.

The *sucA* gene, encoding β-fructofuranosidase, of *R. microsporus* NBRC 32995 was amplified by degenerate PCR, and its complete nucleotide sequence was determined. The results showed that the full-length open reading frame (ORF) was 1590 bp and contained no introns (Figure S1). Next, the deduced amino acid sequence was obtained based on the DNA sequence and subjected to a BLAST homology search. The SucA protein of *R. microsporus* NBRC 32995 exhibited high homology with that of *R. microsporus* ATCC 62417, with an identity of 96%, and also showed homology with that of *R. delemar* NBRC 4754 (80%), *Amylomyces rouxii* CBS 436.76 (77%), *Schwanniomyces occidentalis* ATCC 26077 (62%), and *Debaryomyces hansenii* CBS 767 (61%). The SucA protein has three conserved domains, A, D, and E, belonging to the glycoside hydrolase family [17]. The presence of all domains was confirmed in *R. microsporus* NBRC 32995 (Figure 3). In addition, the amino acid sequence of the A domain in *R. microsporus* NBRC 32995 was closely related to that of *R. delemar* NBRC 4754. Interestingly, the A domain of *R. microsporus* NBRC 32995 includes eight amino acid residues positioned upstream of the MNDPNG sequence that have been deleted from the A domain of *R. microsporus* ATCC 62417 [18] (Figure 3).

**Figure 4:**
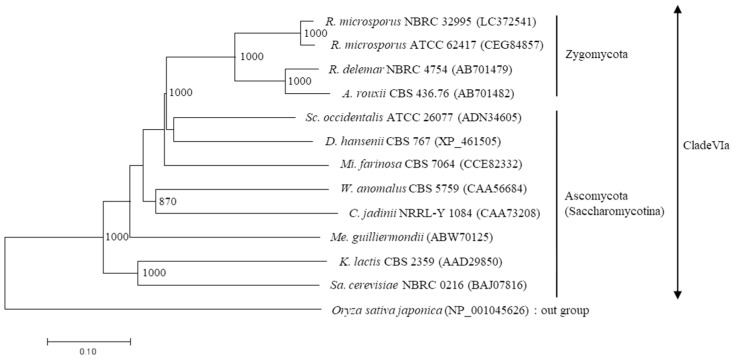
Phylogenetic analyses of the amino acid sequences of microbial β-fructofuranosidases. A dendrogram was constructed using the neighbor-joining method from the amino acid sequences of microbial β-fructofuranosidases belonging to clade VIa. The length of each branch indicates amino acid sequence divergence. Bootstrap values (>800), calculated from 1000 replications, are shown. The numbers in parentheses indicate the accession number.

A dendrogram was created using the neighbor-joining method based on the amino acid sequences of microbial β-fructofuranosidases (Figure 4). This showed that the SucA proteins of *R. microsporus* NBRC 32995 and *R. microsporus* ATCC 62417 belong to clade VIa and are closely related to the β-fructofuranosidases of *R. delemar* NBRC 4754 and *Amylomyces rouxii* CBS 436.76. In addition, while these three *Rhizopus* strains belong to the Zygomycota, their SucA proteins are closely related to the β-fructofuranosidases of *Schwanniomyces occidentalis* ATCC 26077 and *Debaryomyces hansenii* CBS 767, yeasts that belong to the Ascomycota (Saccharomycotina), and are distantly related to *Rhizopus* species (Figure 4).

## 4. Discussion

The results showed that all *Rhizopus* strains tested have the ability to produce organic acids (lactic acid, malic acid, and/or fumaric acid) and/or ethanol in medium with sucrose as a carbon source, indicating that these *Rhizopus* strains are capable of sucrose degradation for the production of organic acids and ethanol. In particular, the levels of these compounds produced by *R. microsporus* NBRC 32995 and *R. stolonifer* NBRC 4781 were significantly higher than those produced by the other *Rhizopus* strains tested and comparable to those produced by *R. oryzae* NBRC 4785 and *R. delemar* NBRC 4754, which were tested previously [7,8]. However, PCR using degenerate primers, which were designed by the conserved sequences of domains A and E in SucA, indicated that DNA amplification occurred only in *R. microsporus* NBRC 32995 among the *Rhizopus* strains tested (Table 1). Additionally, PCR amplification was also conducted using another forward primer designed from the conserved region of domain D in a set of the same reverse primer. The results showed that no amplification occurred for the *sucA* minus strains, which amounted to 11 strains tested in this study. Actually, all *sucA* minus strains in Table 1 did not show sucrose-hydrolyzing activity based on β-fructofuranosidase (data not shown). These results indicated that the *sucA* gene was present only in *R. microsporus* NBRC 32995 among the 12 *Rhizopus* strains tested, and that the *sucA* gene is not common among *Rhizopus* strains (Table 1).

The results of TLC analyses showed that *R. microsporus* NBRC 32995 as well as *R. delemar* NBRC 4754 had the ability to hydrolyze both sucrose and FOS into monosaccharides, and that this process was almost complete by 28 days of incubation. In contrast, *R. oryzae* NBRC 4785 could not hydrolyze FOS (Figure 1). These results support the findings that both *R. microsporus* NBRC 32995 and *R. delemar* NBRC 4754 carry the *sucA* gene, encoding β-fructofuranosidase, while *R. oryzae* NBRC 4785 does not (Table 1). Since both *R. microsporus* NBRC 32995 and *R. delemar* NBRC 4754 produce the SucA protein (β-fructofuranosidase), which degrades FOS from the terminal fructose residue, these results demonstrate that the complete hydrolysis of FOS in these fungal strains is induced by the β-fructofuranosidase secreted from their cells. However, *R. oryzae* NBRC 4785 does not produce this hydrolytic enzyme. Instead, *R. oryzae* is known to degrade sucrose with a glucoamylase, and *R. oryzae* NBRC 4785 was shown to produce glucoamylase in a previous study [7]. The activity of glucoamylase, which degrades FOS from the terminal glucose residue, is inhibited at pH values below pH 4.0 [7]. The pH value in the culture of *R. oryzae* NBRC 4785 was reduced to approximately 3.6 by the release of organic acids following 10 days of incubation (Figure 2C).

The rate of sucrose and FOS degradation by *R. microsporus* NBRC 32995 tended to be lower than that by *R. delemar* NBRC 4754 (Figure 1A–D): while both strains degraded most of the sucrose and FOS in the medium by 10 days of incubation, traces of sucrose at day 10 and FOS at day 21 remained for *R. microsporus* NBRC 32995 (Figure 1A,B). It appears that this result was caused by differences in the sucrose-hydrolyzing activities of the strains. The enzyme activity of *R. microsporus* NBRC 32995 increased during the incubation period and reached approximately 0.1 U/mg protein at day 14 of incubation, but this was approximately 1/4 that of the *R. delemar* NBRC 4754 enzyme (Figure 2A,B). Recently, it has been reported that some *R. microsporus* cells produce thermostable inulinase (EC 3.2.1.7) as a glycolytic enzyme [19], which is secreted as an extracellular enzyme [20]. However, based on the genome database, *R. microsporus* NBRC 32995 does not share genomic sequence homology with genes that encode the inulinase protein, indicating that the hydrolysis of FOS is not caused by inulinase.

According to the dendrogram based on amino acid sequences, enzymes with β-fructofuranosidase activity can be classified into seven clades: the monocotyledonous clades I, II, IV, and V; the dicotyledonous clade III; the fungal clades VIa and VIb; and the bacterial clade VII [9]. In a previous study, the SucA proteins from *R. delemar* and *A. rouxii* were classified into clade VIa [8]. This clade has a conserved sequence of H-[FY]-[ST]-P-x-[KS]-[NG]-[WF]-MNDPNG in domain A and a signal peptide upstream of this region (Figure 3), suggesting the possibility of enzyme secretion outside the cell [9]. The SucA protein of *R. microsporus* NBRC 32995 also contained domain A belonging to clade VIa, and its amino acid sequence was closely related to that of *R. delemar* NBRC 4754 (Figure 3). In addition, the other two highly conserved regions of SucA, domains D and E, were also found in the SucA protein of *R. microsporus* NBRC 32995 and also belonged to this clade. The SucA full-length amino acid sequence of *R. microsporus* NBRC 32995 (530 amino acids) exhibited high homology (approximately 80% identity) with that of *R. delemar* NBRC 4754. These results indicate that the newly identified SucA protein of *R. microsporus* NBRC 32995 should be classified as a clade VIa member (Figure 3 and Figure 4). The amino acid sequences of SucA in the conserved regions of domains D and E were identical between *R. microsporus* NBRC 32995 and *R. microsporus* ATCC 62417. However, an eight-amino acid sequence of HYTPEKGW, positioned upstream of the MNDPNG sequence in domain A of *R. microsporus* NBRC 32995, was deleted in the *R. microsporus* ATCC 62417 sequence (Figure 3), implying that *R. microsporus* ATCC 62417 may not possess proper β-fructofuranosidase activity.

Together, these results suggest that *R. microsporus* NBRC 32995, carrying a full-length *sucA* gene, is a unique strain even among *R. microsporus* and that its *sucA* gene may have been introduced into this strain by horizontal gene transfer. In a previous study, the possibility of the horizontal gene transfer of the *sucA* gene into *Rhizopus* was discussed [8]. Gene transfer is likely to occur through contact with other microorganisms such as yeast during the production of fermented foods [7]. However, it remains unclear whether this is the mechanism by which *R. microsporus* NBRC 32995 acquired its *sucA* gene, as this strain was isolated from chaff and its habitat appears to be distinct from those of other *sucA*-carrying strains, such as *R. delemar* NBRC 4754. Recently, genomic analyses have been conducted for a wide variety of filamentous fungi including *Rhizopus*; thus, the distribution of the *sucA* gene may be further elucidated in the future.

The results obtained in this study show that *R. microsporus* NBRC 32995 is a unique *Rhizopus* strain carrying the full-length amino acid sequence of a SucA protein belonging to clade VIa that is closely related to the SucA proteins of yeasts (Ascomycota), suggesting that other *Rhizopus* species do not have SucA proteins closely related to those of Ascomycota. In addition, *R. microsporus* NBRC 32995 possesses β-fructofuranosidase activity and is capable of hydrolyzing sucrose, along with the production of organic acids (i.e., lactic acid, fumaric acid, and malic acid), even under low pH conditions. Such organic acids are used as raw materials for industrial purposes, such as flavor control in food processing. Thus, *R. microsporus* NBRC 32995 is of great potential value for the simultaneous production of multiple useful organic acids and thus may be useful for industrial applications.

## Figures and Tables

**Table 1:** Detection of *sucA* gene and the production of organic acids and ethanol in *Rhizopus* strains.

Strains		*sucA* ^1^	Concentration (mg/mL)				References
			Malic Acid	Lactic Acid	Fumaric Acid	Ethanol	
*Rhizopus oryzae*	NBRC 4785	−	6.34 ± 1.53	93.67 ± 14.46	0.72 ± 0.45	5.80 ± 1.90	Watanabe and Oda [7]
*Rhizopus delemar*	NBRC 4754	+	34.26 ± 10.03	8.18 ± 1.81	30.28 ± 9.74	30.71 ± 9.36	Orikasa and Oda [8]
*R. microsporus var. chinensis*	NBRC 4737	−	n.d. ^2^	0.25 ± 0.10	n.d.	0.24 ± 0.10	This study
*R. microsporus var. chinensis*	NBRC 4768	−	n.d.	0.11 ± 0.07	n.d.	1.44 ± 0.56	This study
*R. microsporus var. chinensis*	NBRC 31988	−	n.d.	1.00 ± 0.71	n.d.	1.91 ± 0.58	This study
*R. microsporus var. microsporus*	NBRC 32995	+	32.71 ± 6.42	58.06 ± 6.36	7.87 ± 1.41	19.60 ± 0.66	This study
*R. microsporus var. microsporus*	NBRC 32996	−	0.44 ± 0.11	0.31 ± 0.05	n.d.	0.41 ± 0.07	This study
*R. microsporus var. rhizopodiformis*	NBRC 32997	−	n.d.	n.d.	n.d.	0.06 ± 0.01	This study
*R. microsporus var. tuberosus*	NBRC 100014	−	n.d.	0.18 ± 0.07	n.d.	0.15 ± 0.03	This study
*R. stolonifer var. lyococcus*	NBRC 32998	−	2.44 ± 0.58	n.d.	n.d.	0.14 ± 0.04	This study
*R. stolonifer var. stolonifer*	NBRC 4781	−	9.24 ± 1.50	81.26 ± 10.41	5.63 ± 1.32	14.32 ± 1.74	This study
*R. stolonifer var. stolonifer*	NBRC 5411	−	0.93 ± 0.41	0.25 ± 0.06	n.d.	0.23 ± 0.04	This study
*R. stolonifer var. stolonifer*	NBRC 6188	−	1.32 ± 0.41	0.47 ± 0.22	n.d.	2.29 ± 0.39	This study
*R. stolonifer var. stolonifer*	NBRC 30816	−	1.78 ± 0.34	0.26 ± 0.08	n.d.	1.36 ± 0.32	This study

^1^ PCR amplification of *sucA* gene; +, detected; −, not detected. ^2^ not detected.

## Supplementary Materials

The following are available online at http://www.mdpi.com/2076-2607/6/1/26/s1, Figure S1: The full-length open reading frame (ORF) sequence of the *sucA* gene in *R. microsporus* NBRC 32995 (accession number: LC372541).

Supplementary File 1
