# Supplementary material for: Identification of sucA, Encoding β-Fructofuranosidase, in Rhizopus microsporus
Source: Microorganisms. 2018 Mar 13;6(1):26. doi: 10.3390/microorganisms6010026 (PMC5874640; doi:10.3390/microorganisms6010026)
Supplement: Supplementary File 1 [file microorganisms-06-00026-s001.pdf]

```
....|....| ....|....| ....|....| ....|....| ....|....| ....|....| ....|....| ....|....| ....|....| ....|....|
        10         20         30         40         50         60         70         80         90        100
ATGATACAAT TTAAATTAAT ATATATATTT ACATTGATTC AATATGTTTT AGCTAATAAA GCACCTGACA TAAATTCCAT TACAAATAGA CCACTAATTC
....|....| ....|....| ....|....| ....|....| ....|....| ....|....| ....|....| ....|....| ....|....| ....|....|
       110        120        130        140        150        160        170        180        190        200
ATTATACACC TGAAAAAGGT TGGATGAATG ATCCAAATGG TTTATTTTAC GACAAAAAAG ATAAAATTTG GCATTTATAT TTTCAATATA ATCCAAACGA
....|....| ....|....| ....|....| ....|....| ....|....| ....|....| ....|....| ....|....| ....|....| ....|....|
       210        220        230        240        250        260        270        280        290        300
TACTGTTTGG GATTTACCAT TATATTGGGG TCATGCTACT TCAAAAGATT TTGTTAATTG GGAACATCAT GATGTTGCTA TTAGTCCAAA AAATAATAAT
....|....| ....|....| ....|....| ....|....| ....|....| ....|....| ....|....| ....|....| ....|....| ....|....|
       310        320        330        340        350        360        370        380        390        400
GAAGGTATTT ATTCTGGTAG TATTGTAATT GATTACAATA ATACTACTGG TTTTTTCAAT AAATCGATTG ATCCAAATCA AAGGATAGTT GCTATTTATA
....|....| ....|....| ....|....| ....|....| ....|....| ....|....| ....|....| ....|....| ....|....| ....|....|
       410        420        430        440        450        460        470        480        490        500
CTAATAATAT TCCTAATTTG GAAACACAAG ATATTGCTTA TTCTATTGAT GGTGGCTATA CTTTTATAAA ATATGAAAAT AATCCTGTTA TTAATGTTAA
....|....| ....|....| ....|....| ....|....| ....|....| ....|....| ....|....| ....|....| ....|....| ....|....|
       510        520        530        540        550        560        570        580        590        600
TTCCTCTCAA TTTCGTGATC CTAAAGTTTT TTGGCATGAA GAAACTAATC AATGGATTAT GGTTGTTTCA AAATCTCAAG AATATAAAAT TCAAATATTT
....|....| ....|....| ....|....| ....|....| ....|....| ....|....| ....|....| ....|....| ....|....| ....|....|
       610        620        630        640        650        660        670        680        690        700
GGATCCACAA ATTTAAAAAA TTGGAAATTA CACTCCAATT TCACTTCAGG TTATTATGGA TATCAATATG AATGTCCTGG TTTAATTAAA GTTCCAATTG
....|....| ....|....| ....|....| ....|....| ....|....| ....|....| ....|....| ....|....| ....|....| ....|....|
       710        720        730        740        750        760        770        780        790        800
AAAAATCTAG TGAATATAAA TGGGTTATGT TTTTAGCTAT TAATCCGGGT TCACCTATTG GTGGTTCTGC TAATCAATAT TTTATTGGTG ATTTTGATGG
....|....| ....|....| ....|....| ....|....| ....|....| ....|....| ....|....| ....|....| ....|....| ....|....|
       810        820        830        840        850        860        870        880        890        900
ATATAAATTT AAAGTTTATG ATAATCAAAC TAGATTTATG GATTTAGGTA AAGATTTCTA TGCCTTTCAA ACTTTTAGTG ATGTTGATAT ACAAAATCAT
....|....| ....|....| ....|....| ....|....| ....|....| ....|....| ....|....| ....|....| ....|....| ....|....|
       910        920        930        940        950        960        970        980        990       1000
GGTGTTCTAG GTTTAGCTTG GGCATCAAAT TGGCAATATG CCAATAGAGT TCCAGATACA GATAATTATA GAAGTTCAAT GTCTTTGGTT AGAAATTATA
....|....| ....|....| ....|....| ....|....| ....|....| ....|....| ....|....| ....|....| ....|....| ....|....|
      1010       1020       1030       1040       1050       1060       1070       1080       1090       1100
CTTTAGCTTA TGTTCCAAAT AATCCAGAAA CTAAATTGTT AACTTTAATT CAAAAACCTG TTCTTGGAAA TTCATTAAAA ATTAGTAGCA CAATCAATAA
....|....| ....|....| ....|....| ....|....| ....|....| ....|....| ....|....| ....|....| ....|....| ....|....|
      1110       1120       1130       1140       1150       1160       1170       1180       1190       1200
AAAAGATATT GTTATGAATA CCACTACATT CATTGAATTA AAATCTAATT CGATTAATGA TATATTTGAT TTTGATATTA CTTTTAAAGT TATTAATCCA
....|....| ....|....| ....|....| ....|....| ....|....| ....|....| ....|....| ....|....| ....|....| ....|....|
      1210       1220       1230       1240       1250       1260       1270       1280       1290       1300
TCAGTATCTC ATAAAAATAA TTCTAATTTT GATATTATTA TTGGATCACC TAAAAATGAA AATATTAAAT TAGGTTTTGA TCCAGTAGCA CAAGCATTTT
....|....| ....|....| ....|....| ....|....| ....|....| ....|....| ....|....| ....|....| ....|....| ....|....|
      1310       1320       1330       1340       1350       1360       1370       1380       1390       1400
ATGTTGATAG AGGAATTCCT AATAATAAAT TTTATAAAAA TCCATTTTTT ACAGATAAAT TATCAACTTA TCTTGAACCA TTTTCTTATG ACGAAAATAA
....|....| ....|....| ....|....| ....|....| ....|....| ....|....| ....|....| ....|....| ....|....| ....|....|
      1410       1420       1430       1440       1450       1460       1470       1480       1490       1500
TTATGGAAGT TATCAAGTTT ATGGATTAAT TGATAGAAAT ATTATTGAAC TTTATTTTAA CAATGGGTCT ACAACTATGA CAAATACATT TTTCATGAGT
....|....| ....|....| ....|....| ....|....| ....|....| ....|....| ....|....| ....|....| ....|....| ...
      1510       1520       1530       1540       1550       1560       1570       1580       1590
AAAGGAAATT TACCAAACAG TATTAAAATA TTAACATCTA TTGATGATGT ATTTAAAATT GAGGATGCCA ATATTAGAAT ATTATCTTTG TAA
```

Figure S1. The full-length open reading frame (ORF) sequence of the *sucA* gene in *R. microsporus* NBRC 32995 (accession number： LC372541).
